# Hip osteoarthritis: A novel network analysis of subchondral trabecular bone structures

**DOI:** 10.1093/pnasnexus/pgac258

**Published:** 2022-11-21

**Authors:** Mohsen Dorraki, Dzenita Muratovic, Anahita Fouladzadeh, Johan W Verjans, Andrew Allison, David M Findlay, Derek Abbott

**Affiliations:** South Australian Health and Medical Research Institute (SAHMRI), Adelaide, SA 5000, Australia; Australian Institute for Machine Learning (AIML), The University of Adelaide, Adelaide, SA 5000, Australia; School of Electrical and Electronic Engineering, The University of Adelaide, Adelaide, SA 5000, Australia; Centre for Biomedical Engineering (CBME), The University of Adelaide, Adelaide, SA 5000, Australia; Centre for Orthopaedic and Trauma Research, Discipline of Orthopaedics and Trauma, The University of Adelaide, Adelaide, SA 5000, Australia; Centre for Cancer Biology, University of South Australia and SA Pathology, Adelaide, SA 5000, Australia; South Australian Health and Medical Research Institute (SAHMRI), Adelaide, SA 5000, Australia; Australian Institute for Machine Learning (AIML), The University of Adelaide, Adelaide, SA 5000, Australia; Royal Adelaide Hospital, Adelaide, SA 5000, Australia; Adelaide Medical School, The University of Adelaide, Adelaide, SA 5000, Australia; School of Electrical and Electronic Engineering, The University of Adelaide, Adelaide, SA 5000, Australia; Centre for Biomedical Engineering (CBME), The University of Adelaide, Adelaide, SA 5000, Australia; Centre for Orthopaedic and Trauma Research, Discipline of Orthopaedics and Trauma, The University of Adelaide, Adelaide, SA 5000, Australia; Centre for Biomedical Engineering (CBME), The University of Adelaide, Adelaide, SA 5000, Australia; School of Electrical and Electronic Engineering, The University of Adelaide, Adelaide, SA 5000, Australia; Centre for Biomedical Engineering (CBME), The University of Adelaide, Adelaide, SA 5000, Australia

**Keywords:** osteoarthritis, graph theory, networks, machine learning, convolutional neural networks

## Abstract

Hip osteoarthritis (HOA) is a degenerative joint disease that leads to the progressive destruction of subchondral bone and cartilage at the hip joint. Development of effective treatments for HOA remains an open problem, primarily due to the lack of knowledge of its pathogenesis and a typically late-stage diagnosis. We describe a novel network analysis methodology for microcomputed tomography (micro-CT) images of human trabecular bone. We explored differences between the trabecular bone microstructure of femoral heads with and without HOA. Large-scale automated extraction of the network formed by trabecular bone revealed significant network properties not previously reported for bone. Profound differences were discovered, particularly in the proximal third of the femoral head, where HOA networks demonstrated elevated numbers of edges, vertices, and graph components. When further differentiating healthy joint and HOA networks, the latter showed fewer small-world network properties, due to decreased clustering coefficient and increased characteristic path length. Furthermore, we found that HOA networks had reduced length of edges, indicating the formation of compressed trabecular structures. In order to assess our network approach, we developed a deep learning model for classifying HOA and control cases, and we fed it with two separate inputs: (i) micro-CT images of the trabecular bone, and (ii) the network extracted from them. The model with plain micro-CT images achieves 74.6% overall accuracy while the trained model with extracted networks attains 96.5% accuracy. We anticipate our findings to be a starting point for a novel description of bone microstructure in HOA, by considering the phenomenon from a graph theory viewpoint.

Significance Statement In this paper, we have explored a novel network analysis methodology for describing microcomputed tomography images of human trabecular bone. In addition, we have explored differences between the trabecular bone microstructure of femoral heads with and without hip osteoarthritis, with the aim of improved quantitative understanding of the underlying characteristics of those differences.

## Introduction

Hip osteoarthritis (HOA) is a degenerative hip joint disease that leads to progressive damage of articular cartilage (1), and structural changes underlying the subchondral bone that clinically manifest with overall changes in the (i) shape of the femoral head, (ii) loss of joint space, (iii) frequent severe pain, and loss of joint function (2). Consequently, HOA is considered as a major cause of disability and loss of life quality (3), with a prevalence of around 10% for people above 65, where 50% of these cases are symptomatic (4). With an aging global population, the prevalence of osteoarthritis (OA) is continuing to increase (5), and is associated with escalating healthcare costs (6). Thus, optimal management of HOA is of vital importance, which relies on an improved understanding of the underlying factors for disease initiation and progression.

It has become clear that events in the subchondral bone under the articular cartilage are intimately involved in the development of OA (7). In particular, it is well-documented that HOA involves pathological changes in the trabecular bone of the femoral head (8, 9). The sequence by which these abnormalities contribute to disease initiation and progression and how they develop, has not been elucidated (10).

The subchondral bone together with articular cartilage forms an “osteochondral” functional unit with its primary role to maintain joint function. Once synergy between cartilage and subchondral is disrupted, significant structural changes occur in the whole joint. The application of high-resolution imaging approaches such as computed tomography (CT) and magnetic resonance imaging (MRI), in the evaluation of OA patients, has enabled detection of specific OA tissue characteristics in the osteochondral unit and expands the possibilities for diagnosis of disease at an early stage. Of the particular interest are structural changes within subchondral bone. Both animal and human studies indicate that changes of subchondral bone take place early and may precede changes in the articular cartilage and, thus it may contribute to not only the initiation but progression of disease (11).

Subchondral bone immediately beneath cartilage comprises two parts: the subchondral plate and subchondral trabeculae. The subchondral plate is formed by a thin layer of dense bone, from which arise thin trabeculae, forming an intricate and complex network of trabecular bone. Subchondral sclerosis, which encompasses thickening of both the subchondral plate and subchondral trabeculae, is commonly observed in advanced OA and is, thus considered as a hallmark of OA (12). However, it has been reported that in the various stages of OA, different microstructural changes of subchondral bone occur, and it depends on the distance from the articular surface (13–17). For example, in both humans and animal models of disease during early stages of OA, elevated bone remodeling, subchondral bone plate thinning, and increased porosity were shown to be significantly correlated with cartilage damage (13). Also during the late stage of OA, appositional bone tissue growth is noted and results in increased subchondral bone apparent density, thickening of the subchondral bone plate, increased bone volume, decrease of trabecular separation, increased trabecular thickness, and change of trabeculae from rod-like into plate-like structures, which associate temporally with articular cartilage thinning and deterioration (10). Regardless of elevated bone volume density, subchondral bone is less stiff, less mineralized, and less able to withstand repetitive loading (18).

First, described by Wolff (19), the bone undergoes dynamic bone remodeling to adapt to the loads, to which it is subjected, and to maintain structural and mechanical integrity. Later, Radin and Rose proposed that an increase in bone density may potentially lead to elevated stiffness that introduces unfavorable stress concentrations resulting in damage to the cartilage (20, 21). More recently, it was suggested that conventional assessments such as thickening of trabeculae, bone volume density, and changes of and thickening of trabeculae structure model index can be insensitive to more subtle changes, and that application of modern image analytical approaches such as individual trabecula segmentation (ITS), are necessary for detection of more early and subtle changes in the properties of the subchondral trabecular bone structure in health and disease (22–24).

A machine learning approach was proposed to assess the ability of semiautomatically extracted MRI-based radiomic features from tibial subchondral bone to distinguish between knees without and with OA. Although the approach was able to classify OA and normal cases, it was not able to explain the link between individual radiomic features and OA (25).

Also, a deep learning approach was used for grading HOA features on radiographs. In a similar way with previous approach, this study used machine learning as a “black box” classifier and it was not able to elucidate the relation between OA and visual features in radiographs (26).

Here, for the first time we propose a novel “network” approach to analyze the trabecular structure based on the concepts of graph theory in the human femoral head in subjects with advanced HOA and we made comparisons with age matched control bone with no history of bone disease. Then we develop a machine learning model to classify HOA and control cases. In contrast to previous machine learning approaches, our network approach is now able to elucidate the link between the incidence OA and network visual features that demonstrate for the first time.

There are several examples of biological networks, such as metabolic networks, protein interaction networks, neural networks, and vascular networks (27–30). Several complex systems have been investigated recently from a network viewpoint that links the different elements comprising them (31). The development of tumor vascular networks has recently been explored via graph theory (32). Network analysis can qualitatively and quantitatively reveal vital information on the unique characteristics of biological phenomena (33–36). The question we explore here is: can trabecular bone microstructure of the femoral head, described as a network graph, visualized by a group of vertices and edges, distinguish between control and HOA bone? Addressing this question may lead to the development of a framework for understanding the progression of the abnormalities in HOA.

We find that a network approach can accurately distinguish between trabecular bone of the femoral head and HOA disease from control bone, despite the wide variability of presentation of trabecular bone between HOA samples. The extraction of the trabecular architecture obtained from microcomputed tomography (micro-CT) imaging into a mathematical graph maps the data from a high dimensional space into a low dimensional space. Using a machine learning model, we demonstrate that the low dimensional network representation retains meaningful properties of the original data, near to its intrinsic dimension.

## Results

### OA structural abnormalities assessed by micro-CT

**Fig. 1.**
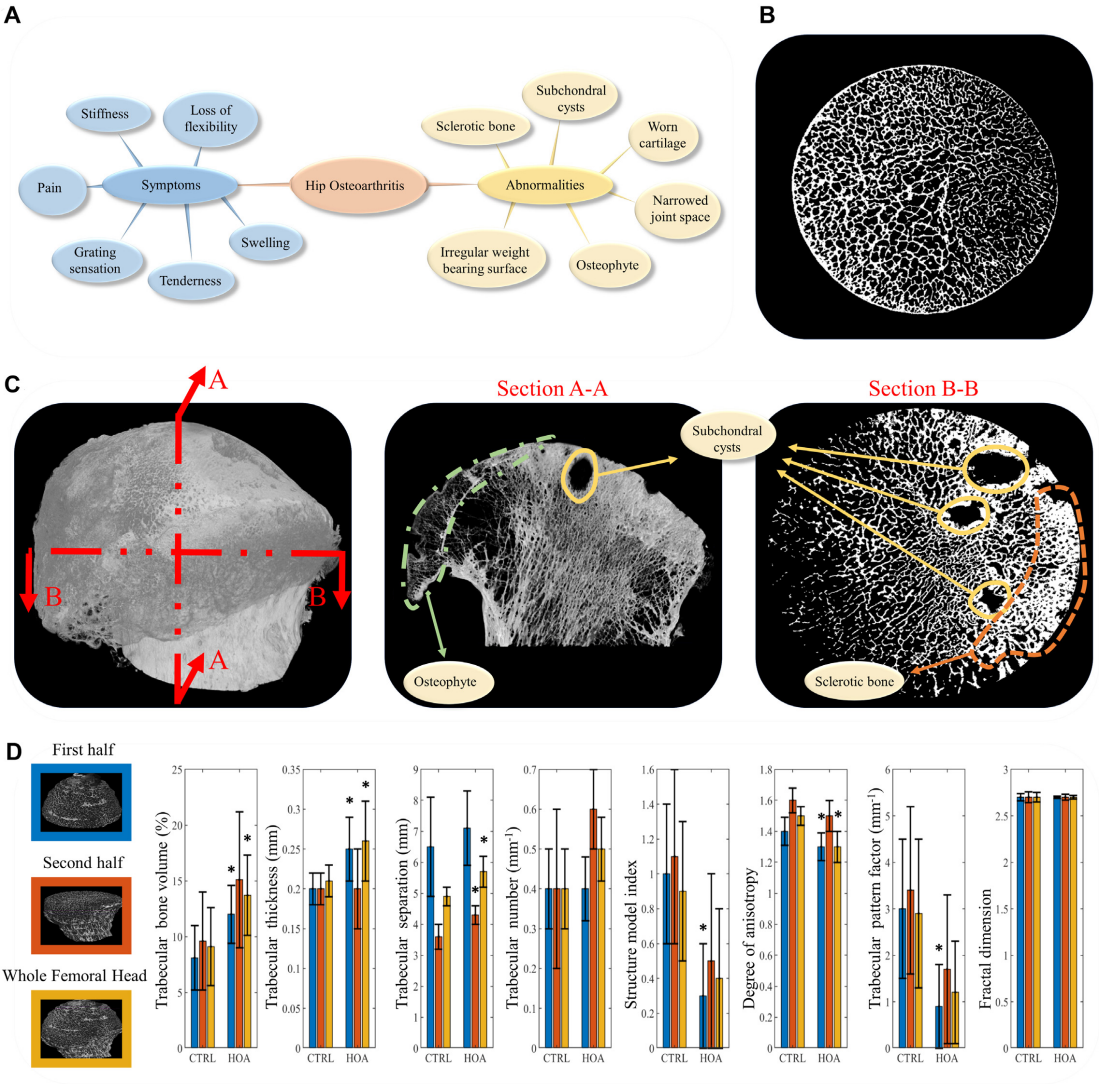
Femoral head abnormalities in HOA. (A) HOA symptoms and abnormalities are shown. (B) A representative micro-CT image of trabecular bone in control femoral head without bone-related diseases and (C) micro-CT images from A–A and B–B sections corresponding to a HOA patient illustrate the abnormalities in trabecular bone. (D) An analysis of micro-CT images obtained from a cohort of seven patients with HOA and a cohort of seven CTRLs shows the parameters of trabecular thickness, trabecular bone volume, structural model index, trabecular separation, trabecular number, degree of anisotropy, trabecular bone pattern factor, and fractal dimension. Means and SDs are shown on the bars for the first half (blue), second half (orange), and whole femoral head (yellow). Here, * indicates statistical significance (*P* < 0.05) between CTRL and HOA. The comparison among the group were performed using unpaired t test.

In this study, high-resolution ex vivo imaging using micro-CT provided an opportunity to study the microstructure in the trabecular bone of the femoral head in HOA patients, compared with unaffected hips. Macroscopically, the HOA femoral heads showed considerable variability, qualitatively and in terms of disease severity. The general characteristics, as shown in Fig. 1(A), were described on the basis of damage of cartilage (loss of cartilage volume and integrity), and development of subchondral bone sclerosis, osteophytes, and bone cysts. A representative micro-CT image of control trabecular bone is shown in Fig. 1(B), while 1(C) shows images of micro-CT slices of an HOA femoral head.

The femoral heads obtained from subjects undergoing hip replacement surgery and from control (CTRL) individuals without a history of bone disease were scanned using micro-CT (Skyscan 1076, Kontic, Belgium, isotropic resolution = 20.5 μm). After acquiring the micro-CT images, the numerical (trabecular number, bone volume fraction, thickness, and separation) and topographic (degree of anisotropy, structural model index, and trabecular pattern factor) properties of subchondral bone microstructure were assessed. Using NRecon software, v2.0.4.2, data were analyzed separately for three volumes of interest: whole sample (total of 1,950 images = 40 mm), first half of the specimen (images 0 to 975 = 20 mm), the proximal portion of the femoral head, which articulates with the acetabulum of the pelvis (severely affected by OA-related changes such as loss of cartilage integrity, presence of subchondral bone cyst), and the second half (images 976 to 1,950 = 20 mm), the distal portion, toward femoral neck (relatively intact cartilage and no subchondral bone degradation).

The results presented in Fig. 1(D) for the first half (blue bars), the second half (orange bars), and whole femoral head (yellow bars) indicate that HOA trabecular bone had increased bone volume, due mainly to an increased trabecular thickness compared to controls. A similar finding was seen by comparing the trabecular thickness and separation, comparing whole volumes of the femoral head. Trabecular separation is described as the thickness of the spaces measured after image binarization. In this study, the increase of trabecular separation in HOA samples is consistent with the presence of numerus bone cysts present in volume of interest. By contrast, the HOA trabecular bone showed decreased topographical properties, such as structure model index, degree of anisotropy, and trabecular pattern factor, compared to controls. The structure model index shows the relative prevalence of plates and rods. Isotropy is an indication of 3D symmetry or the presence or absence of preferential alignment of structures through a specific directional axis. Trabecular pattern factor is a measure of connectivity, where a highly disconnected trabecular structure is indicated with a higher trabecular pattern factor. The results did not show any significant difference for fractal dimension between HOA and CTRL cases. Fractal dimension is a measure of surface complexity of an object, that quantifies how an object's surface fills space.

Although these parameters provide useful information about the whole femoral head, they are volume-based parameters and do not shed light on the effects of HOA at different depths. In addition, the large SDs in Fig. 1(D) indicate the presence of uncertainty as the data points are far from the mean. Therefore, defining a more accurate framework that can reveal the differences caused by HOA, across the depth of the sample, is desirable.

### HOA: a network analysis

**Fig. 2.**
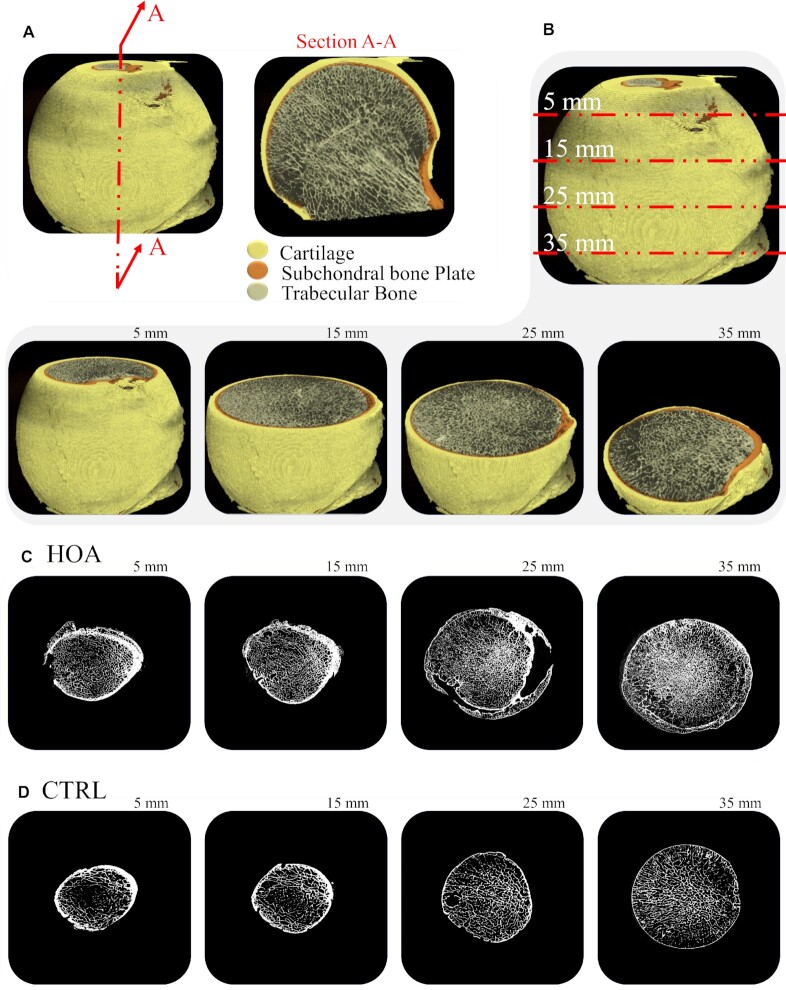
Representative 3D model of subchondral bone changes as a function of depth. (A) A section of hip bone showing cartilage, subchondral bone, and trabecular bone. (B) Four sections at various depths, 5, 15, 25, and 35 mm, are considered, and the corresponding (C) HOA and (D) CTRL micro-CT images are shown. Although the CTRL trabecular structure appears smoother, the network pattern in both cases changes as a function of depth.

Next, we examined the trabecular networks imaged as micro-CT image slices, obtained from the HOA and CTRL femoral heads. A section of hip bone indicating cartilage, subchondral bone and trabecular bone from proximal (top) to distal (bottom) is shown in Fig. 2(A). As described previously, we found that HOA trabecular bone was structurally disordered; this was mainly due to increased trabecular thickness. We analyzed the trabecular bone in HOA and CTRL groups as a set of networks and investigated the behavior of networks as a function of bone depth. As an example, four sections at various depths (5, 15, 25, and 35 mm) are shown in Fig. 2(B), and the corresponding HOA and CTRL micro-CT images are shown in Fig. 2(C) and (D). It may be seen that the CTRL trabecular structure appears more uniformly distributed and that the network pattern in both cases changes as a function of depth.

**Fig. 3.**
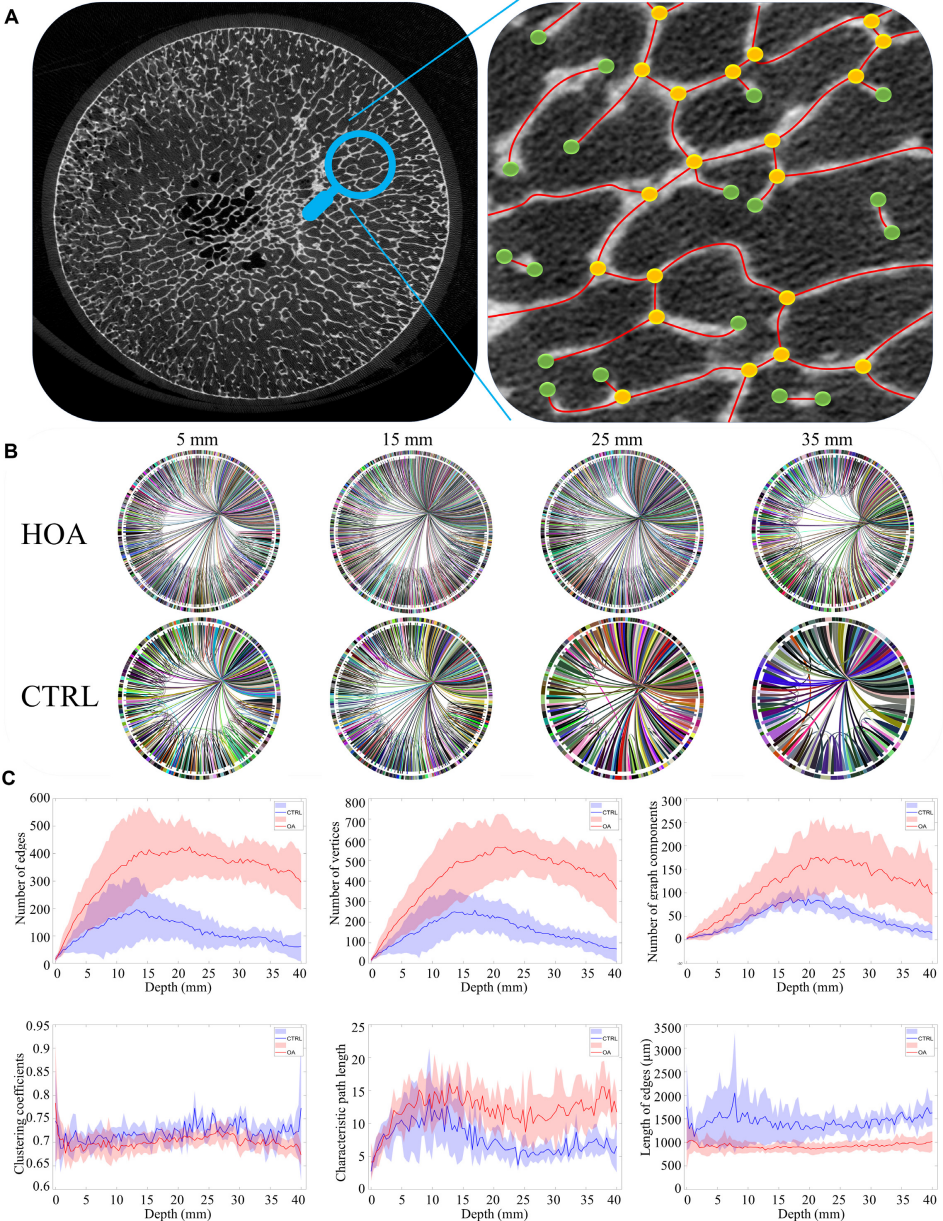
HOA network analysis. (A) An example of identifying edges and vertices from micro-CT images showing networks in HOA (seven femoral heads) and CTRL (seven femoral heads). (B) Circular layout of HOA and CTRL corresponding to an HOA and a CTRL case (shown previously in Fig. 2(C) and (D) demonstrating a reducing number of vertices and edges at various depths. (C) We analyzed micro-CT images obtained from seven HOA (red) and seven CTRL (blue) cases and calculated mean (shown with solid lines) and SD (shaded areas). The corresponding number of vertices, number of edges, number of graph components, clustering coefficients, characteristic path length, and length of edges show the evolution of the bone network in HOA and CTRL cases as a function of depth.

To investigate the trabecular bone network in HOA samples, we developed customized image processing software using MATLAB that is able to extract 2D networks in micro-CT images and display the vertices, edges, and several graph parameters in the networks. As an example, in Fig. 3(A), a micro-CT image is shown on the left side, and the extracted edges (red lines), vertices (yellow nodes), and branches (green nodes) are illustrated on the right. To further understand the network behaviors, the networks for a HOA and a CTRL case were “regraphed,” and the networks were visualized using circular layouts, as shown in Fig. 3(B). It can be seen that the entire number of vertices and edges relating to the HOA network is greater than for CTRL, indicating the formation of compressed trabecular structures.

Complex biological networks are often characterized by nonlinearly interacting parameters. To study this intricate connectivity, we further investigated several network parameters, in particular number of vertices, number of edges, number of graph components, clustering coefficients, characteristic path length, and length of edges in Fig. 3(C). To achieve this, we analyzed micro-CT images from HOA (red) and CTRL (blue) groups—solid lines and shading indicate mean and SD, respectively.

For the micro-CT slices close to the top of the femoral head, the number of vertices and edges grows faster for HOA cases; however, when they reach the maximum point at the first third (top) of the femoral head, both HOA and CTRL decline gradually at a similar rate. These observations show that although HOA trabecular bone has a greater number of vertices and edges, the network in CTRL bone is more connected, as HOA possesses more graph components than CTRL. Notable also was a similar trend in the clustering coefficients in HOA and CTRL; however, it may be seen that there is a minor increase in the HOA clustering coefficients. The characteristic path length in HOA is greater than CTRL and both show a fluctuating pattern. The shorter length of edges in HOA indicates the impact of the abnormalities caused by sclerotic changes, increased anisotropy, and elevated plate/rod ratio.

The method given here for presenting HOA and CTRL micro-CT images via a network framework can be integrated potentially into machine learning models for HOA diagnosis. As future intelligent disease diagnosis relies on optimizing machine learning methods and focusing on data-centric approaches, our network representation shows promise as an effective feature for perform machine learning classification.

### Network properties: effective feature for machine learning models

To evaluate the outcome of network analysis, we employed a machine learning model for classifying HOA and CTRL. We developed a deep convolutional neural network (CNN) and fed it with two separate inputs (i) micro-CT images of the trabecular bone, and (ii) the network extracted from the images.

With the advent and progress of AI, researchers have been attempting to employ deep neural networks as a novel approach for diagnosis based on clinical and medical data. As machine learning is evidence-based and can analyze problems in an unbiased way, it can be helpful for making objective diagnosis from biomarkers or clinical data (37, 38). Among the deep learning architectures, CNNs are particularly suitable for medical image classification due to their ability to take advantage of natural image properties, such as shared weights, local connections, and pooling. They employ several layers of feature detectors to perform preferred analysis (31) and also they are able to perform data processing tasks in the form of multiple layers.

Several CNN architectures are used widely for medical image diagnosis, including mitosis detection in breast cancer (39), deformable registration of MR brain images (40), and classification of skin cancer (41). Recently, the advent of machine learning systems has attracted the attention of OA researchers. A classification approach based on a probabilistic neural network (PNN) classifier was proposed for the characterization of hips from pelvic radiograph images as OA or normal (42). Textural features in this study were extracted from X-ray images. The model accomplishes a high classification rate; however, a limitation is that it lacks automated performance, as it needs a manual procedure via a graphics cursor for recognizing regions of interest (ROI) in the X-ray images.

**Fig. 4.**
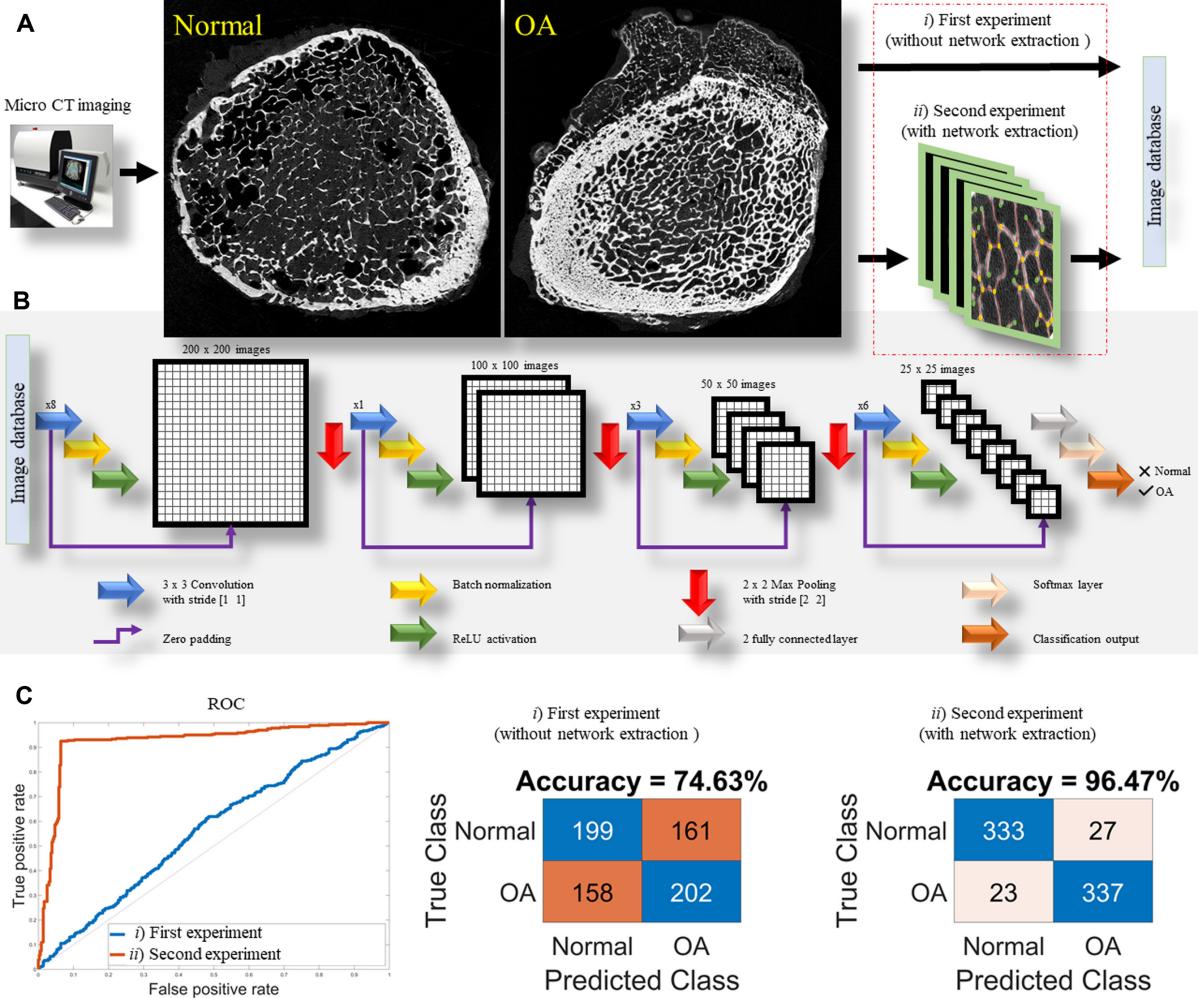
Machine learning model overview. (A) The system includes micro-CT imaging and a pipeline of preprocessing techniques fed separately with raw images and extracted networks. (B) Schematic of the CNN operation. It comprises four blocks, each of which are made of a 3 × 3 convolution layer with stride [1 1], a batch normalization and finally a ReLU function. Also, three max pooling layers with stride [2 2] reduce the size of images is used in this architecture. It is followed by two fully connected layers, a Softmax layer and a classification output. (C) Here, a ROC curve for the approach is shown in blue (without network extraction) and red (with network extraction) lines, where the second experiment outperforms the first at the task of classification. In addition, the confusion matrices are illustrated for both experiments.

We designed a multiple layer CNN architecture and trained it on 90% of our dataset. Here, Fig. 4 shows the architecture of the proposed CNN deep network. To evaluate the network analysis, we trained the model first with the micro-CT images, and then assessed the classification output. Then, in a separate procedure, we fed the model with the extracted networks. The database contains (i) 360 OA and 360 control micro-CT images that are composed of human expert-labeled images, and (ii) the corresponding networks. We divided the dataset under analysis into training (90% of images) and test (10% of images) sets. After training the deep CNN, we validated the performance of the approach using the test data. The CNN trained on plain micro-CT images achieved 74.6% overall accuracy while the CNN trained on extracted networks attained 96.5% accuracy on a subset of the test set. Here, Fig. 4 summarizes our results for CNN model during both experiments.

## Discussion

In this study, we investigated a novel approach for analyzing the microstructure of the trabecular bone in HOA, compared with healthy control bone, from a graph theory perspective. By applying our software to high-resolution micro-CT images to analyze the entire trabecular microstructure at various depths, we discovered significant differences in the network configuration in HOA compared with controls that were not apparent using conventional outcome measures.

Imaging approaches such as MRI and radiography have been used widely to diagnose HOA (43). Unlike plain radiographs, CT can quantify femoral and acetabular forms and evaluates anatomic relationships independently of patient position, and through several postprocessing methods is able to render a 3D visualization of the hip that simplifies observation of bone morphological features. The CT scan provides an accurate assessment (44) of the bone anatomy of the hip with a high accuracy of 1° to 4.5°. Despite all these advances in imaging technology, how these OA abnormalities change as a function of depth into trabecular bone was unknown until now.

The development of high-resolution imaging, along with the advent of powerful computational approaches, has recently attracted the attention of HOA researchers due to their power in detecting early alternation in bone structure. The imaging technologies including peripheral quantitative computed tomography (pQCT), MRI, dual-energy X-ray absorptiometry (DXA), and micro-CT have revealed the main evidence that elucidates the significant role of trabecular bone in the pathogenesis of HOA. The high-resolution micro-CT used for the present ex vivo study exceeds the resolution provided by current clinical imaging. Clinically used CT scanning works in a similar manner but with lower resolution. However, pQCT scanning is rapidly approaching a resolution sufficient for a version of the analysis described here, suggesting that detection of bone abnormalities at early stages and improved understanding of heterogeneity may soon be possible, and importantly offering the potential for early identification and treatment of these abnormalities. This in turn may potentially lead to attenuation of cartilage damage and the prevention of OA progression (45–48).

To understand more fully the changes that take place in the subchondral bone in HOA, we assessed the network properties within bone microstructure of femoral head of HOA cases and CTRL bone. Our results show important changes in trabecular bone parameters, with increases in mean trabecular thickness, bone volume, and trabecular separation in HOA, and a concomitant decrease in degree of anisotropy, trabecular pattern factor, and structure model index. The novel feature of this study is the inclusion of network parameters. Our results show that the most significant network changes in HOA such as the number of edges, vertices, and graph components appear in first third (most proximal) of the femoral head (from top to 1,300 µm), and after that both HOA and CTRL networks show similar behavior. This suggests that subchondral bone changes relate to the OA disease and do not pre-exist the disease. We suggest that these changes and abnormalities in trabecular bone proximal to the diseased joint will open up new avenues for designing artificial intelligence (AI) approaches for HOA diagnosis and classification.

Our analyses reveal that HOA networks are characterized by reduced length of edges, indicating the compressed trabecular structures in patients with HOA. Also, HOA networks show elevated characteristic path lengths and reduced clustering coefficients, therefore, lowering the small world network characteristics as they evolve. A network possesses small world properties where its characteristic path length is relatively low, and where its clustering coefficient is relatively high (49). A high clustering coefficient occurs with highly connected groups, while a short mean path length occurs with rapid information spread (50–52).

Additionally, we demonstrated the effectiveness of deep learning for HOA classification via plain micro-CT images and the extracted networks. Using a CNN trained on both datasets, we have shown that employing our network approach for feature extraction can significantly increase the classification accuracy. Although we highlight that clinical HOA diagnosis is based on several factors beyond the morphological features in CT scan images, the ability to diagnose OA with high accuracy has the potential to enable earlier intervention and to expand access to vital medical care.

We anticipate our findings to be a starting point for a more sophisticated description of bone microstructure in HOA, by considering the phenomenon from a graph theory viewpoint. Network-based quantitative measures potentially open new avenues for assessing trabecular bone microstructure at other skeletal sites and in other skeletal disease states.

Because OA etiology is likely multifactorial, and comprises multiple phenotypes, in this study only subjects with primary radiographic and symptomatic OA were included to ensure that detected changes in subchondral trabecular bone are related to this disease. Several diseases such as: OA, rheumatoid arthritis, osteoporosis, and psoriatic arthritis are characterized with severe structural changes in subchondral bone. Note that the subchondral bone changes in all these diseases are elicited mainly due to altered bone turnover but their manifestations are various from bone erosion to bone sclerosis (53).

An interesting open question, for future study, is to analyze the number and characteristics of branches in the network to check for correlation with bone resorption.

One of the limitations in this study is that the model did not assess other abnormalities that may coexist with HOA. However, in this study our focus was to investigate and document if a network representation can be used to inform us about bone changes beyond increase/decrease of bone trabecular volume and bone mineral density in the whole femoral head rather than with small volumes of interest that are often used in animal and/or human studies. Therefore, this method may potentially be adapted and used in future clinical HOA studies to benefit medical practice.

A second limitation of our study regards the small number of samples. Although the number of samples in this study was sufficient for statistical investigation and building network analysis, more examples are needed to prove the generality of our findings and to increase the accuracy of our machine learning model. This study is also a cross-sectional study and could not evaluate how the bone structure changes from early stage to late-stage HOA disease. In general, it is challenging to analyze bone microstructure in early-stage HOA patients by micro-CT, since the bone samples are not accessible and/or cadaveric tissue with early HOA changes is not easily available. Thus, it will eventually be of clinical interest to investigate a larger cohort longitudinally to determine if our method might play a useful role in assessment of bone across disease severity and/or may help to differentiate OA from other diseases of the joint.

In addition, clinical use of ultrahigh (7 Tesla) MRI has improved sensitivity for detection of pathologies within subchondral bone such as bone marrow lesions, and early changes within bone microarchitecture. Thus, we believe that the network analysis presented in this study will be a promising pathway for future determination of the role of subchondral bone in progression of OA and an early detection of changes within subchondral bone characteristic for humans OA disease.

Moreover, parameters describing subchondral trabecular bone structure in HOA (bone volume fraction, trabecular bone pattern factor, trabecular number, trabecular thickness, trabecular separation, and structure model index) that are often described by micro-CT are already adapted and evaluated in vivo using multidetector row CT (54–56). More recently, it was elegantly demonstrated that use of clinical CT is a new and promising imaging tool in clinical assessment of both hip and knee OA (57–59). Thus, we believe that our method will further enrich current knowledge about subchondral bone involvement in pathogenesis of HOA.

In summary, the goal of the paper was to show that the trabecular structure forms a network that can be mathematically characterized, and this is our new fundamental result. It is well-known in the machine learning community that if data has too many features (ie. high dimensionality) the amount of training data required exponentially increases. This is a well-known problem called the curse of dimensionality, coined by Richard E. Bellman (60). One of the steps toward robust machine learning is to first preprocess the data to reduce its dimensionality so that only essential features are classified. By extracting network parameters of the trabecular structure and using this reduced data, we are in fact reducing dimensionality as desired. Consequently, our paper shows that classification accuracy is 74.6% and this significantly increases to 96.5% when we reduce dimensionality as described.

## Materials and Methods

### Human material

A total of seven femoral heads (four males and three females aged 71.7 ± 14.6) were collected from patients who have undergone total joint replacement for late-stage HOA at the Royal Adelaide Hospital and seven cadaveric femoral heads (five females and two males aged 65.8 ± 15) accessed through the SA Tissue Bank, SA Pathology, Royal Adelaide Hospital Mortuary.

Inclusion criteria for HOA subjects were radiographic HOA with severe symptomatic disabilities including limited mobility and severe pain. Inclusion criteria for nondisease subjects were no evidence of radiographic HOA or joint pain in medical history. Exclusion criteria for both groups: osteoporosis, rheumatoid arthritis, metabolic bone disease, history of malignancy, and medication that may have affected bone turnover. Written consent was obtained for all subjects and the study received prior approval from the Human Research Ethics Committee.

### Data collection

To collect the data related to the microstructure of the trabecular bone, whole femoral head samples were scanned using a micro-CT scanner (Skyscan 1276, Skyscan-Bruker, Kontich, Belgium). Scanner settings: 20.5 μm isotropic pixel size, source voltage 100  kVp, current 200 μA, rotation step 0.4°, 180° rotation, exposure time 700 mm, and 3-frame averaging.

For an individual femoral head this generated 3,305 X‐ray projection images (826 projections per step), image resolution was 3,872 × 3,872 pixels (79,453 × 79,453 µm) in size with depth resolution of 20 µm, in 16‐bit Tiff format, producing a total dataset of 24.3 GB, scan duration approximately 4 h.

To ensure adjustment for clustering (multiple regions of interest) and impact of age and sex, the data were analyzed in a series of separate linear mixed effects regression models to control the confounding of age and sex as independent variables.

### Statistical analysis

The Shapiro–Wilk test was employed to test normality of the data distribution. Differences between groups (control vs. HOA) were described using the unpaired t test. We performed the statistical analyses via GraphPad Prism software (Version 9.2.0 for MacOS). Data is reported as the mean ± SD. The critical value of *P* < 0.05 was chosen for significance.

### Deep learning model

Our CNN model shown in Fig. 4(B) contains four blocks, each of which comprise a 3 × 3 convolution layer with stride [1 1], a batch normalization layer and finally a ReLU function. In addition, three max pooling reduces the size of images, and the network followed by two fully connected layers, a Softmax layer and finally a classification output.

The layers where filters are applied to the original image are called convolutional layers. Batch normalization is applied to the output of the previous layers allowing every layer of the network to learn more independently. It can be considered as regularization to avoid overfitting of the model. We used a ReLU activation function to control the output. Note that ReLU is a linear function that will output the input directly when it is greater than zero, otherwise, it forces a zero output.

In Fig. 4(C), a receiver–operator characteristic (ROC) curve and confusion matrices were obtained as model metrics. A ROC curve is a graph illustrating the performance of a classification model at all classification thresholds. The confusion matrix is a table with two rows and two columns that reports FN, TP, TN, and FP.

Here, FN represents false negative, an incorrect prediction in the negative class; TP represents true positive, a correct prediction in the positive class; TN is true negative, a correct prediction in the negative class; and FP represents false positive, an incorrect prediction in the positive class.

### The network analysis software

The trabecular structures of the bone were extracted using our custom MATLAB software. This computational approach, based on image processing tools, assists in extracting useful information from trabecular networks, avoiding miscalculation. Therefore, we developed software that receives the network images and precisely outputs a number of useful parameters such as number and position of lines and junctions, histogram, graph parameters, and so on.

The algorithm consists of the following steps:

Step (1) Reading image: read in the bone micro-CT images.
Step (2) Image adjusting: the RGB images are converted to grayscale and image intensity values or colormap is adjusted to improve image contrast. In addition, the area of interest is segmented, and any object out of border of area can be removed.
Step (3) B/W filtering: the grayscale images converted to B/W, and after image enhancement, the small holes in vessel images can be removed. In addition, the scatter points related to a few cells that are not connected to tubular structures may be removed.
Step (4) Outline extraction: using this morphological operation, all objects are reduced to lines in 2D binary images.
Step (5) Finding an individual line: trabecular junctions are extracted, and the lines between them identified as an individual ridges. This information assists to calculate the graph parameters. We provide open access to this software for use by future researchers through https://github.com/Dorraki/Bone

### Small-worldness, clustering coefficient, and path length

In a network consisting of *N* vertices, the distance *L_ij_* between two vertices, *n_i_* and *n_j_* is given by the length of the shortest path between the vertices, that is, the minimal number of edges that need to be traversed to travel from vertex *n_i_* to *n_j_*. The average or characteristic path length *L* = < *L_ij_* > of a network is defined as the average distance between all pairs of vertices. The clustering coefficient relates to the local cohesiveness of a network and measures the probability that two vertices with a common neighbor are connected. In the case of undirected networks, given a vertex *n_i_* with *k_i_* neighbors, there exist *E_max_ = k_i_* (*k_i_ −* 1)/2 possible edges between the neighbors. The clustering coefficient *C_i_* of the vertex *n_i_* is then given as the ratio of the actual number of edges *E_i_* between the neighbors to the maximal number *E*_max_, therefore, *C_i_* = 2*E_i_/k_i_*(*k_i_ −* 1). Small-worldness can be achieved via both characteristic clustering coefficient (*C*) and path length (*L*) with respect to a single reference graph: σ = *CC_r_/LL_r_*, where *C_r_* and *L_r_* are the mean clustering coefficient and characteristic path length for an equivalent random network, respectively (32).

## Data Availability

The study data are included in the article. The code generated for this work has been deposited on GitHub: https://github.com/Dorraki/Bone.
